# Development of a Logistic Regression Model for Colorectal Cancer Relapse Prediction in Sabah, Malaysia

**DOI:** 10.1007/s13193-025-02476-5

**Published:** 2025-11-25

**Authors:** Melvin Ebin Bondi, Syed Sharizman bin Syed Abdul Rahim, Richard Avoi, Mohd Firdaus bin Mohd Hayati, Mohd Hanafi bin Ahmad Hijazi, Romnalin Keanjoom

**Affiliations:** 1 Department of Public Health Medicine, Faculty of Medicine and Health Sciences, Universiti Malaysia Sabah, Kota Kinabalu, Sabah Malaysia; 2 Department of Surgery, Faculty of Medicine and Health Sciences, Universiti Malaysia Sabah, Kota Kinabalu, Sabah Malaysia; 3 Faculty of Computing and Informatics, Universiti Malaysia Sabah, Kota Kinabalu, Sabah Malaysia; 4 Division of Community Health, Faculty of Public Health, Naresuan University, Phitsanulok, Thailand

**Keywords:** Colorectal cancer, Relapse prediction, Logistic regression, Case-control study, Malaysia, Survivorship, Risk stratification

## Abstract

Colorectal cancer (CRC) remains one of the leading causes of cancer-related deaths in Malaysia, with relapse contributing substantially to poor survival outcomes. Despite advances in treatment, relapse surveillance continues to rely on non-individualized schedules. This study aimed to develop and internally validate an interpretable logistic regression model for predicting relapse among Malaysian CRC survivors using routinely available clinical and pathological variables. A retrospective case-control study was conducted using data from hospital-based cancer registries and oncology records across selected public hospitals in Sabah. Patients diagnosed between 2015 and 2020 who completed curative-intent treatment with at least five years of follow-up were included. Ten routinely collected clinicopathological variables were evaluated as candidate predictors using multivariable logistic regression. Model performance was assessed with 10-fold cross-validation. Discrimination was measured using the area under the receiver operating characteristic curve (AUC), and calibration was assessed across risk strata. The optimal probability threshold was identified using the Youden index. Six predictors remained significant in the final model, including tumor stage, lymphovascular and perineural invasion, carcinoembryonic antigen level, tumor grade, and completeness of chemotherapy (*p* < 0.02). The final model demonstrated strong discrimination (AUC = 0.85, 95% CI 0.81–0.89) with good calibration (Hosmer–Lemeshow *p* = 0.42). Sensitivity and specificity were balanced, and internal validation confirmed model stability across folds. Predicted probabilities were stratified into low, moderate, and high-risk categories to support tailored follow-up planning. This validated model offers a clinically practical approach to relapse risk stratification among Malaysian CRC survivors. Its reliance on routinely available data ensures scalability, particularly in resource-limited settings. Integration into the digital CARE-CRC tool may enable risk-adapted surveillance and improve long-term outcomes within Malaysia’s public healthcare system.

## Introduction

Colorectal cancer (CRC) remains one of the leading causes of cancer morbidity and mortality in Malaysia [9, 19]. It ranks among the top three cancers nationally and continues to contribute significantly to cancer-related deaths despite advances in screening and treatment [3, 4, 9]. The Malaysia National Cancer Registry reports that the five-year survival rate for CRC remains below 50%, with relapse accounting for a major proportion of late mortality [19]. Relapses place an additional strain on oncology services, particularly in Sabah, where treatment centers are limited to only two [2] referral centers and caters 3.4 million populations in Sabah [27]. Follow-up logistics and geographical barriers in Sabah, together with the absence of risk-adapted surveillance tools in routine practice, complicate survivorship care delivery [12, 13, 18].

Surveillance following curative treatment is a key element of CRC survivorship care [16, 21]. However, current practice in Malaysian hospitals employ uniform, time-based follow-up schedules that do not account for individual variation in relapse risk [18, 21]. This approach may lead to both inefficient use of limited oncology resources and missed opportunities for early relapse detection [18, 19]. Regional evidence suggests that risk-adapted surveillance tools can improve detection efficiency and reduce healthcare burden, enabling earlier intervention when relapses occur [2, 5, 16]. Programs in Singapore and Thailand have shown that risk-stratified follow-up allows better resource allocation and earlier identification of recurrent disease [16].

Predictive models provide an evidence-based framework for tailoring follow-up intensity, but most relapse prediction tools are derived from Western countries and depend on molecular or radiomic data that are rarely available in Malaysian settings [1, 15, 30, 31]. A locally validated, clinically interpretable model based on routinely collected variables is therefore needed [17, 18]. Logistic regression remains particularly suitable for this purpose where predicting outcomes is the goal because it is transparent, statistically stable, and readily integrated into existing hospital data systems [26, 28, 29].

Within this context, the present study aimed to develop and internally validate a logistic regression model to predict relapse among Malaysian CRC survivors using accessible clinicopathological variables. The ultimate goal was to support risk-adapted surveillance consistent with the objectives of Malaysia’s National Strategic Cancer Control Program (2021) and the Health White Paper [17, 18] and strengthen survivorship care within resource-limited healthcare settings.

## Materials and Methods

### Study Design and Setting

This retrospective case-control study was conducted in selected public hospital in Sabah, Malaysia, which serve as regional referral centers for colorectal cancer management. Data was retrieved from Surgical Clinic, Queen Elizabeth Hospital, Sabah (for relapse cases) as active patients’ data were kept and stored there, while for non-relapse cases were sourced from the NCD Sector, Sabah State Department (the main source of data collection in the state of Sabah). The study aimed to develop and internally validate a logistic regression model to predict colorectal cancer relapses using routinely collected clinicopathological variables, following recognized methodological frameworks for prediction model development [7, 26, 28]. Observation endpoints were fixed at five years after completion of curative-intent treatment for both relapse and non-relapse groups to harmonize follow-up time. Observational reporting also adhered to STROBE recommendations for case-control studies.

## Study Population and Eligibility Criteria

Patients with the first histologically confirmed diagnosis of colorectal cancer between January 2015 and December 2020 were screened for eligibility. 642 patients were screened where inclusion criteria included completion of curative-intent treatment, availability of at least five years of follow-up data, and documented relapse status. Patients were excluded if they had hereditary colorectal cancer syndromes, incomplete clinical records, or were lost to follow-up. 142 patients were excluded making it only a total of 500 patients who have met the criteria and recruited in this study.

## Case and Control Definition and Matching Strategy

Relapses were defined as patients who developed loco-regional or distant recurrence after completion of curative treatment. Controls were relapse-free patients with comparable follow-up duration. A frequency-matching approach was applied, in which relapse and non-relapse controls were matched by year of diagnosis to ensure comparable follow-up time and reduce surveillance bias. A 1:1 ratio (250 cases and 250 controls) was used to enhance statistical efficiency and maintain adequate power for logistic regression analysis [26, 28]. Controls were randomly selected from eligible relapse-free patients who fulfilled the same inclusion criteria.

## Data Sources and Data Collection

Data was extracted from hospital-based cancer registries, oncology records, operative notes, histopathology reports, record units and follow-up clinic documentation. All data were checked for completeness and consistency before analysis. Analyses used complete-case data after excluding records with missing values for outcome or key predictors.

## Outcome Definition

The primary outcome was colorectal cancer relapses, defined as radiological or histologically confirmed recurrence occurring after completion of curative-intent treatment.

### Predictor Variables

Ten routinely available clinicopathological variables were initially evaluated as candidate predictors based on established literature and clinical relevance [15, 30, 31]. These included demographic, tumor-related, and treatment-related characteristics frequently associated with colorectal cancer outcomes. Univariable logistic regression was first performed to screen candidate variables, and those with p-values less than 0.20 were subsequently considered for inclusion in the final multivariable model, consistent with standard model-building approaches [28]. The specific variables and their univariable results are presented in the results section.

## Sample Size Justification

The study evaluated ten candidate predictors for potential inclusion in the multivariable model. We followed contemporary guidance for minimum sample size in prediction modelling for binary outcomes, targeting at least 10 events per candidate parameter to reduce overfitting and ensure precise estimation [26]. Based on Sabah registry experience, this study anticipated a five-year relapse proportion of approximately 50% among CRC survivors, which supported a balanced case-control sample of 250 relapse and 250 non-relapse patients. The dataset included 250 relapse cases and 250 non-relapse controls, exceeding this recommended threshold and providing sufficient statistical power for model development and internal validation.

## Model Development

Multivariable logistic regression analysis was conducted using a forced-entry approach, with all predictors retained after univariable screening entered simultaneously in the final model. This study assessed multicollinearity using variance inflation factors, considering VIF greater than 5 as evidence of problematic collinearity. The variable ‘metastasis at presentation’ showed high collinearity with pathological stage and was therefore excluded from multivariable modelling. Adjusted odds ratios (AOR) with 95% confidence intervals (CI) were calculated to estimate the strength of association between predictors and relapse. Reporting follows the TRIPOD statement for prognostic model development and validation [7].

### Internal Validation

Internal validation was performed using 10-fold cross-validation. The dataset was randomly partitioned into 10 equal subsets. The model was trained on nine subsets and tested on the remaining subsets. This process was repeated ten times, and performance metrics were averaged to generate pooled estimates of model discrimination and calibration [28].

### Model Performance Evaluation

Discrimination was assessed using the Area Under the Receiver Operating Characteristic Curve (AUC). Calibration was evaluated by comparing predicted and observed probabilities across risk groups. Sensitivity, specificity, positive predictive value, and negative predictive value were calculated at the optimal cut-point to assess classification performance. The optimal probability threshold was identified using the Youden index at 0.48 cut points [28, 29]. All reported metrics were averaged across folds to provide pooled estimates.

### Risk Stratification

Predicted relapse probabilities were used to classify patients into low, moderate, and high-risk categories to support risk-adapted follow-up strategies.

### Pilot Evaluation

A pilot usability evaluation was conducted among 20 clinicians involved in colorectal cancer care. A five-point Likert scale was used to assess usability, comprehensibility, perceived clinical utility, and overall satisfaction with the risk-stratification output.

## Results

**Fig. 1:**
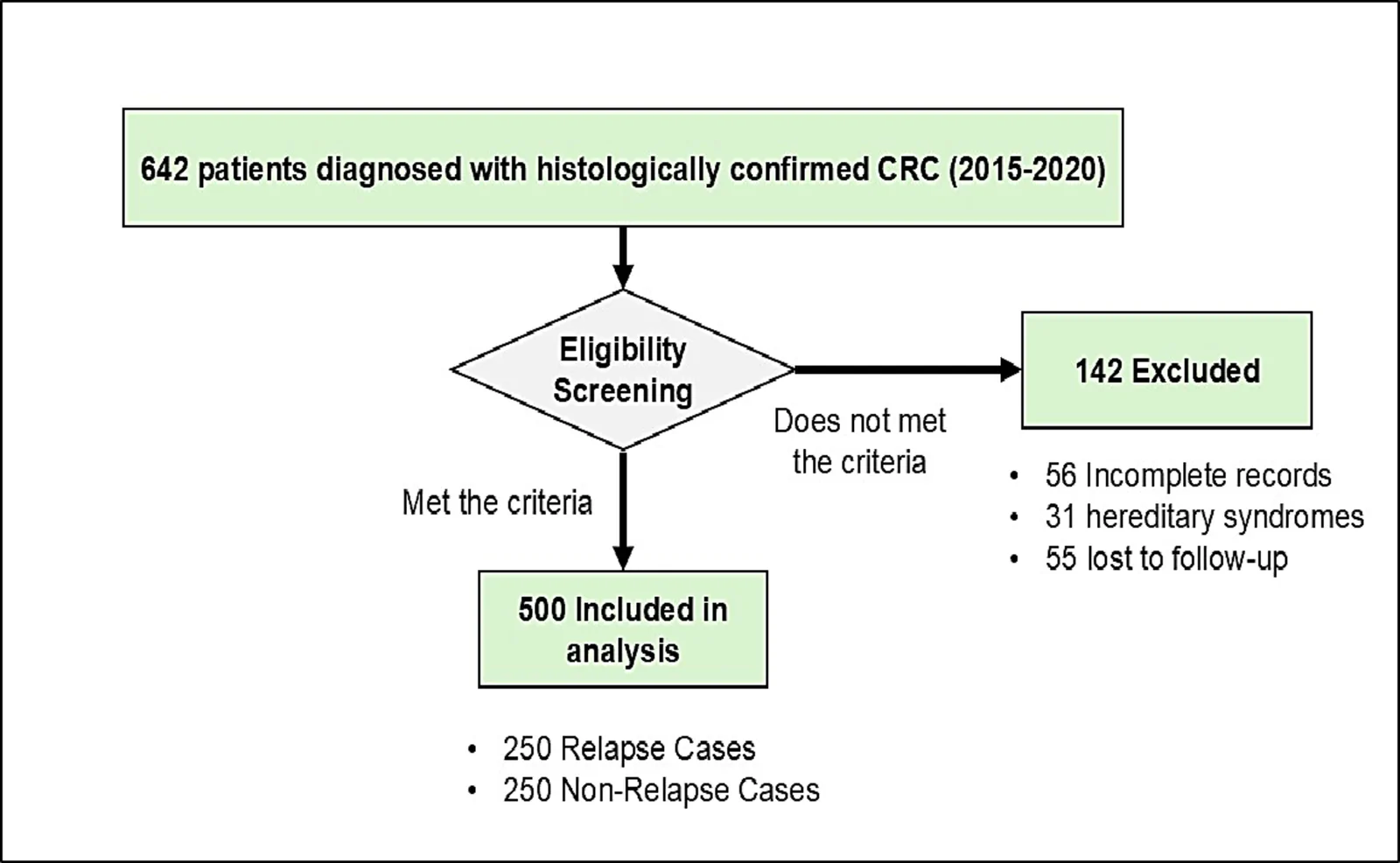
Flowchart of patient selection

A total of 642 patients with histologically confirmed colorectal cancer diagnosed between 2015 and 2020 were screened for eligibility. After excluding 142 patients (56 incomplete records, 31 hereditary syndromes, 55 lost to follow-up), 500 patients were included in the final analysis (250 relapse cases and 250 non-relapse controls) as shown in Fig. 1.

### Baseline Characteristics

The demographic and clinicopathological characteristics of all 500 patients are summarized in Table 1. The mean age was 58.6 ± 11.4 years, and 54% were male. The majority of cases were adenocarcinomas of the colon (68%). Stage III and IV disease were more common among relapse cases, while early-stage disease predominated in controls. Lymphvascular invasion, perineural invasion, elevated carcinoembryonic antigen (CEA), and high tumor grade were all observed more frequently among patients who experienced relapse.

**Table 1:** Baseline characteristics of patients with and without relapse (*n* = 500)

Variable	Relapse (*n* = 250)	Non-relapses (*n* = 250)	*p*-value
Age, mean ± SD (years)	58.9 ± 11.6	58.3 ± 11.3	0.612
Male sex, n (%)	138 (55.2)	132 (52.8)	0.631
Tumor site, colon n (%)	173 (69.2)	167 (66.8)	0.572
Histological subtype, adenocarcinoma n (%)	235 (94.0)	232 (92.8)	0.612
Stage III–IV, n (%)	181 (72.4)	98 (39.2)	< 0.001
Lymphovascular invasion, n (%)	164 (65.6)	96 (38.4)	< 0.001
Perineural invasion, n (%)	141 (56.4)	77 (30.8)	< 0.001
Elevated CEA (> 5 ng/mL), n (%)	154 (61.6)	84 (33.6)	< 0.001
High tumor grade, n (%)	121 (48.4)	71 (28.4)	< 0.001
Incomplete chemotherapy, n (%)	112 (44.8)	53 (21.2)	< 0.001

### Univariable Logistic Regression

Ten candidate predictors were examined using univariable logistic regression (Table 2). Higher tumour stage, lymphovascular invasion, perineural invasion, elevated pre-treatment CEA, high tumour grade, and incomplete chemotherapy were significantly associated with increased odds of relapse. Age, sex, tumour site, and histological subtype did not show significant associations at the 0.05 level but were included in screening to maintain analytic transparency.

**Table 2:** Univariable logistic regression for predictors of colorectal cancer relapses

Predictor	Unadjusted OR	95% CI	*p*-value
Age (years)	1.01	0.99–1.03	0.431
Male sex	1.09	0.77–1.55	0.621
Tumor site (colon vs. rectum)	1.11	0.74–1.67	0.618
Histological subtype (adenocarcinoma vs. others)	1.34	0.68–2.64	0.402
Stage III–IV	3.92	2.73–5.63	< 0.001
Lymphovascular invasion	2.99	2.06–4.33	< 0.001
Perineural invasion	2.86	1.99–4.11	< 0.001
Elevated CEA (> 5 ng/mL)	3.19	2.21–4.60	< 0.001
High tumor grade	2.36	1.61–3.44	< 0.001
Incomplete chemotherapy	3.01	2.02–4.47	< 0.001

### Multivariable Logistic Regression Model

Six predictors remained in the final multivariable logistic regression model (Table 3). Advanced stage at diagnosis (AOR = 2.78; 95% CI 1.95–3.97), lymphovascular invasion (AOR = 2.24; 95% CI 1.56–3.21), perineural invasion (AOR = 1.87; 95% CI 1.22–2.85), elevated CEA (AOR = 2.12; 95% CI 1.48–3.04), high tumour grade (AOR = 1.76; 95% CI 1.11–2.81), and incomplete chemotherapy (AOR = 3.41; 95% CI 2.11–5.52) were independently associated with relapse risk. Age and sex were screened in univariable analyses but were not retained in the final multivariable model due to lack of independent association after adjustment.

**Table 3:** Multivariable logistic regression model predicting colorectal cancer relapse

Predictor	Adjusted OR (AOR)	95% CI	*p*-value
Stage III–IV	2.78	1.95–3.97	< 0.001
Lymphovascular invasion	2.24	1.56–3.21	< 0.001
Perineural invasion	1.87	1.22–2.85	0.004
Elevated CEA (> 5 ng/mL)	2.12	1.48–3.04	< 0.001
High tumour grade	1.76	1.11–2.81	0.017
Incomplete chemotherapy	3.41	2.11–5.52	< 0.001

### Model Validation and Performance

**Fig. 2:**
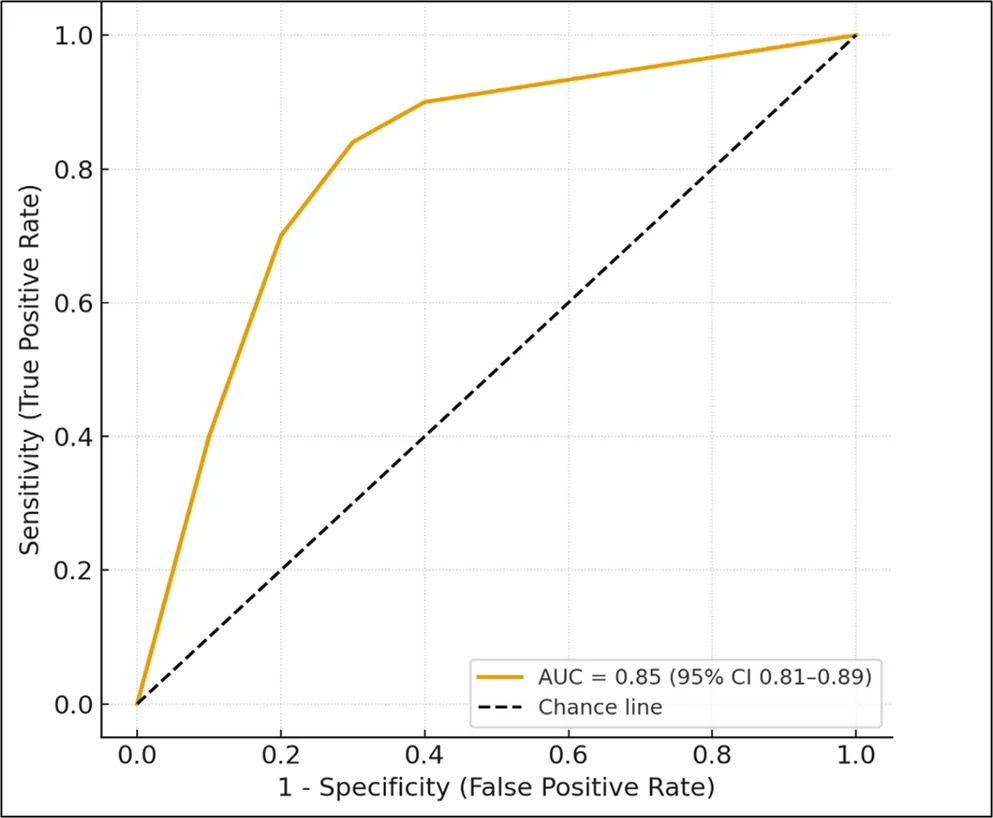
ROC curve showing model discrimination (AUC = 0.85, 95% CI 0.81–0.89) Footnote : Hosmer–Lemeshow test, *p* = 0.42. AUC = 0.85 (95% CI 0.81–0.89)

**Fig. 3:**
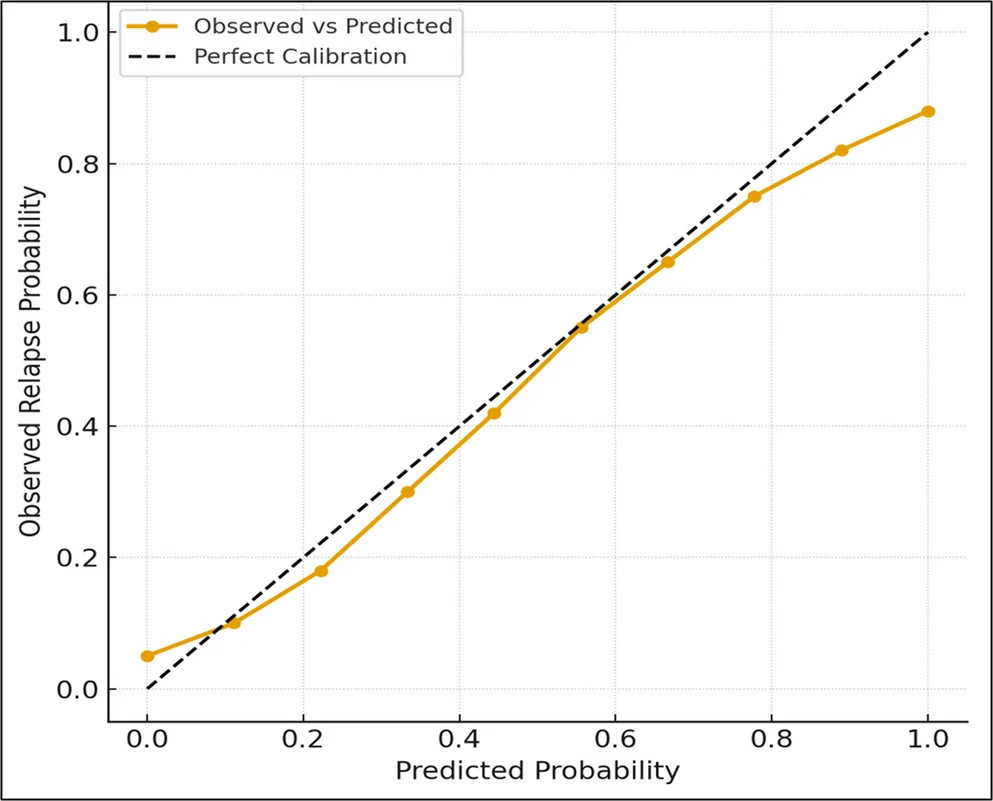
Calibration plot between predicted and relapsed probability

Internal validation using 10-fold cross-validation demonstrated consistent predictive performance. The mean area under the receiver operating characteristic (ROC) curve was 0.85 (95% CI 0.81–0.89), indicating excellent discrimination (Fig. 2). The Hosmer–Lemeshow goodness-of-fit test was non-significant (*p* = 0.42), suggesting good calibration between predicted and observed relapse probabilities (Fig. 3). The model achieved an overall accuracy of 82%, sensitivity 84%, specificity 80%, positive predictive value 83%, and negative predictive value 81% at the optimal probability threshold of 0.48.

### Risk Stratification

**Fig. 4:**
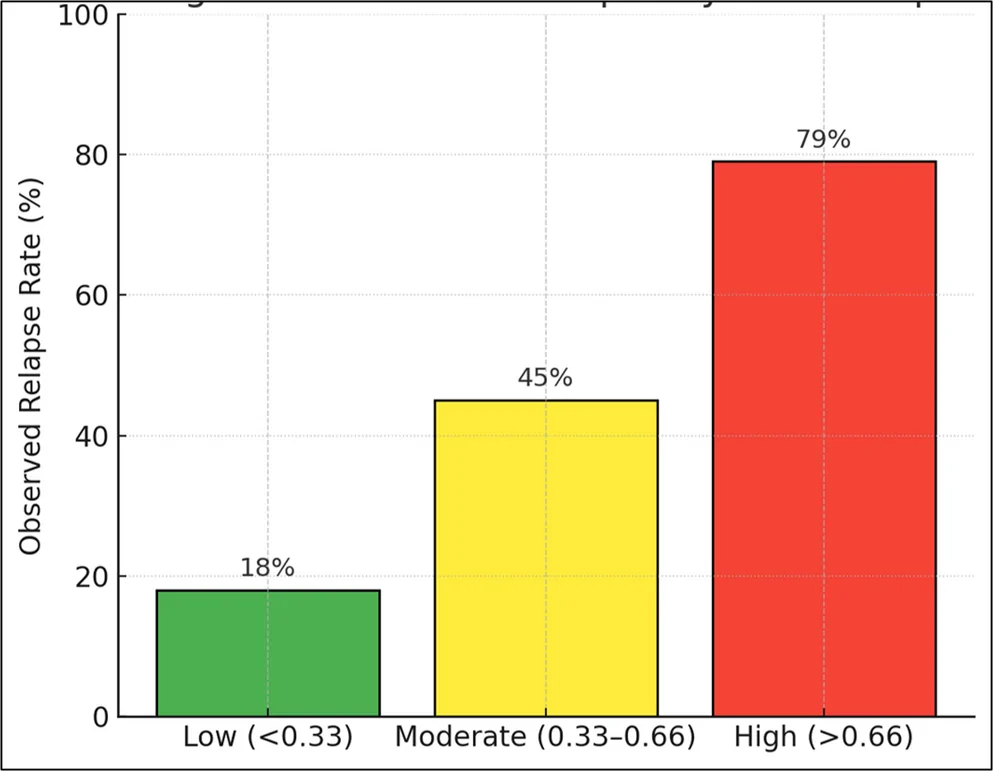
Observed relapse by risk group

Predicted relapse probabilities (Table 4) were used to classify patients into three risk categories: low (< 0.33), moderate (0.33–0.66), and high (> 0.66). Approximately 40% of patients fell into the low-risk group, 37% into the moderate-risk group, and 23% into the high-risk group. The observed relapse rates increased across categories with 18%, 45%, and 79%, respectively, demonstrating effective discrimination between risk strata as shown in Fig. 4.

**Table 4:** Risk stratification output

Risk group	Predicted probability	Patients, *n* (%)	Observed relapse (%)
Low	< 0.33	200 (40.0)	18
Moderate	0.33–0.66	185 (37.0)	45
High	> 0.66	115 (23.0)	79

### Pilot Evaluation Results

20 clinicians, including oncologists, surgeons, and medical officers, participated in the pilot usability evaluation. The mean Likert score was 4.6 ± 0.4 across domains of usability, comprehensibility, perceived clinical utility, and satisfaction (Table 5). Participants reported that the risk stratification output was easy to interpret and could support prioritization of follow-up scheduling and patient counselling.

**Table 5:** Pilot usability evaluation among clinicians (*n* = 20)

Evaluation Domain	Mean ± SD	Median (IQR)	Interpretation
Usability	4.7 ± 0.4	5 (4–5)	Excellent
Comprehensibility	4.6 ± 0.5	5 (4–5)	Excellent
Perceived clinical utility	4.5 ± 0.4	4 (4–5)	Very good
Overall satisfaction	4.6 ± 0.3	5 (4–5)	Excellent

## Discussion

This study established a clinically interpretable model for predicting colorectal cancer relapse using six standard clinical and pathological features commonly recorded in Malaysian hospitals. The model demonstrated robust internal validity and aligns with international findings that key pathological factors remain strong relapse indicators even in diverse populations [15, 31]. In a context like Sabah, where oncology care faces logistical constraints, such a locally calibrated model holds practical value for guiding patient follow-up intensity and improving the allocation of limited resources [18, 27].

### Interpretation of Predictors

Advanced tumor stage continues to represent one of the most reliable determinants of recurrence risk [31]. Patients diagnosed at later stages often harbour residual microscopic disease, making relapse more likely despite curative surgery [14]. Lymphovascular and perineural invasion both signal biological aggressiveness; they facilitate tumour cell dissemination through vascular and neural pathways and have been consistently associated with earlier recurrence and poorer survival [6, 31]. Elevated carcinoembryonic antigen levels before treatment similarly reflect systemic disease activity and have proven prognostic value in numerous cohorts [15, 30, 31]. High tumour grade indicates cellular dedifferentiation and correlates with faster progression, while incomplete chemotherapy highlights a modifiable, health-system factor that often reflects disparities in access or adherence to treatment protocols [8, 17, 18]. These variables together represent a pragmatic balance between biological and service-related predictors, offering a model that is both evidence-based and implementable in Malaysian settings.

#### Context Within Malaysian and Regional Evidence

Relapse patterns in Malaysia resemble those seen across Asia, where resource variation influences both detection and outcomes [16, 19]. National data suggest that only about half of colorectal cancer survivors achieve five-year survival, with relapse contributing substantially to late mortality [19]. The present model, trained on Sabah data, complements these observations and introduces a reproducible way to stratify follow-up intensity within existing Ministry of Health frameworks [18]. Similar risk-stratified approaches in Singapore and Thailand have reduced unnecessary imaging and clinic visits while improving early relapse detection [16]. This underscores how locally validated predictive models can support equitable, data-driven survivorship care [17, 18].

### Model Choice and Clinical Interpretability

Logistic regression was intentionally chosen for its transparency and ease of interpretation [28]. In environments where complex algorithms are impractical, models must be explainable to the clinicians who apply them [29]. While newer machine-learning techniques can achieve marginally higher discrimination, they often require extensive datasets and computational infrastructure unavailable in most Malaysian centers [1, 10, 11]. Logistic regression enables clinicians to interpret how each factor contributes to relapse probability and to communicate risk meaningfully to patients [28, 29, 32]. This emphasis on explainability aligns with World Health Organization guidance on the ethics and governance of AI for health [22].

#### Implications for Surveillance Practice

Current post-treatment surveillance in Malaysia is largely uniform, guided by time-based protocols rather than patient-specific risk [18, 21]. Integrating this model into survivorship care would allow clinicians to tailor follow-up intensity. High-risk patients could be prioritized for closer imaging and CEA monitoring, whereas low-risk individuals could continue with standard review intervals [21]. This stratification supports the goals of the National Cancer Control Program [18] to deliver efficient, risk-adapted care while reducing hospital congestion and cost burden. In practical terms, implementation could begin through web-based dashboards or integration into hospital registry systems, enabling automatic generation of risk scores at the end of treatment [20, 22–25]. This framework is being operationalized through the web-based CARE-CRC Tool, developed by the authors under Universiti Malaysia Sabah, which incorporates this logistic regression model to generate individual relapse-risk scores for clinical decision support.

#### Strengths, Limitations, and Future Work

A major strength of this study is its grounding in real-world clinical data and the use of variables that require no specialized assays or imaging. This study has also several important covariables that were missing, such as gender, BMI status, history of cancer, comorbidities, smoking, drinking, history of hormonal use. These are frequently reported in the literature as predictors and suggested to be included in future study. The internal validation adds methodological rigor, ensuring reliability across patient subgroups [7, 26, 28]. Nonetheless, the retrospective design may introduce residual confounding, and single-region data limit generalizability to other Malaysian states [28]. The model also omits molecular markers that could enhance prediction, though such tests are rarely accessible outside tertiary centers [15, 30]. Relapse status among excluded patients with incomplete records could not be ascertained, which may introduce selection bias. Detailed timestamps for time-to-event were incomplete across hospitals, preventing survival analysis and potentially attenuating predictors whose effects vary with time. Future work should involve multicenter validation and temporal calibration to assess reproducibility, followed by external validation in broader ASEAN datasets. Integration with electronic medical records and mobile platforms will be a necessary next step for real-time clinical application [12, 20].

## Conclusion

This model offers a practical, interpretable framework for relapse risk prediction among colorectal cancer survivors in Malaysia. It bridges statistical rigor with clinical usability, aligning with national priorities for personalized, data-driven cancer surveillance. Continued validation and digital implementation could transform it into a scalable tool for improving outcomes in colorectal cancer survivorship.

## Data Availability

The datasets generated and analyzed during the current study are available from the corresponding author upon reasonable request.
